# Dental treatment and caries prevention preceding treatment under general anaesthesia in healthy children and adolescents: a retrospective cohort study

**DOI:** 10.1007/s40368-018-0332-1

**Published:** 2018-02-26

**Authors:** M. Grindefjord, J. Persson, L. Jansson, G. Tsilingaridis

**Affiliations:** 1 Department of Pediatric Dentistry, Public Dental Health Services, Eastmaninstitutet, Dalagatan 11, 113 24 Stockholm, Sweden; 2 Division of Pediatric Dentistry, Department of Dental Medicine, Karolinska Institutet, Stockholm, Sweden; 3 Center of Pediatric Oral Health, Stockholm, Sweden; 4 Division of Periodontology, Department of Dental Medicine, Karolinska Institutet, Huddinge, Sweden; 5 Department of Periodontology, Public Dental Health Services, Eastmaninstitutet, Stockholm, Sweden

**Keywords:** Caries, Prevention, Children, General anesthesia, Adolescents

## Abstract

**Aim:**

This was to examine healthy children and adolescents treated under general anaesthesia (GA) and a matched control group not receiving GA to compare treatment and preventive care received prior to GA treatment.

**Methods:**

This retrospective cohort study included 71 healthy subjects and 213 age- and gender-matched control subjects. The treatment group had been consecutively referred from the Public Dental Health Service (PDS) in Stockholm to the Department of Paediatric Dentistry, Eastman Institute, Stockholm during 2006–2007. Data was extracted from the patient records at the PDS, including variables such as number of dental visits, treatment/prophylaxis prior to GA, number of missed and cancelled appointments, and number of decayed teeth.

**Results:**

On average, the treatment group had significantly more decayed teeth (*p* < 0.001) than the control group. Furthermore, the treatment group had significantly more restorations (*p* < 0.01), had visited the dentist significantly more often (*p* < 0.001), and had undergone significantly more behaviour management treatment and preventive treatment (*p* < 0.001). In the treatment group 65% of the children and adolescents, had received no behaviour management treatment and 48%, no preventive treatment.

**Conclusions:**

In the Stockholm PDS, over half of the children and adolescents referred by general dentists to paediatric specialists had no behaviour management treatment and nearly half, no preventive treatment, despite receiving significantly more operative treatment compared with matched controls. General dentists should target high caries-risk patients for additional behaviour management and preventive care to reduce the need for treatment under GA.

## Introduction

There are approximately two million children and adolescents in Sweden aged 0–19 years, and their oral and dental health has improved over the years. According to the Swedish National Board of Health and Welfare, caries prevalence has reduced to less than half that of two to three decades previously (National Board of Health and Welfare 2008). However, in some risk groups, such as immigrant children and children living in areas of lower socio-economic status, caries remains a major problem (Grindefjord et al. 1995; Stecksén-Blicks et al. 2014).

In Sweden, 10% of all children referred to specialists in paediatric dentistry receive treatment under general anaesthesia (GA) (Klingberg et al. 2010). The most common are the need for major treatment, dental fear, problems related to chronic illness or disability, and for young children, a lack of cooperation with dental treatment (Klingberg et al. 2010). For adolescents, untreated severe dental caries is largely a consequence of long-term avoidance of dental care (Skaret et al. 2004; Jamieson et al. 2009).

The aetiology of dental caries is multifactorial, such as oral hygiene habits, dietary habits, and fluoride intake (Mejàre et al. 2014). Tooth decay in both the primary and permanent dentitions can cause pain due to either infection or treatment, and pain is a strong predictor for developing dental fear and/or dental avoidance (Skaret et al. 1998; Skaret et al. 1999; Low et al. 1999). The most important reasons for using GA, as reported by parents, are dental fear and repeated unpleasant experiences during dental treatment (Savanheimo et al. 2005). Uncooperative children with severe caries pose a demanding challenge to Public Dental Health Service (PDS). Savanheimo and Vehkalahti (2008) reported that early identification of high caries risk patients and intensive preventive care are the key to reducing the number of children receiving treatment under GA due to severe dental caries. Treating children and adolescents with severe dental decay represents a failure in dental prevention. The aim of this study was to compare healthy children and adolescents treated under GA with a healthy age- and gender-matched control group not receiving GA, in order to evaluate operative and preventive treatment given prior to GA.

## Methods

**Fig. 1:**
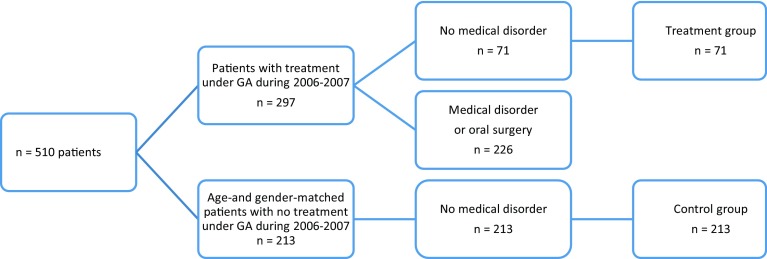
Flowchart of patient recruitment

This retrospective cohort study comprised healthy patients referred from the PDS in Stockholm to the Department of Paediatric Dentistry, Eastman Institute, Stockholm between January 2006 and October 2007 and a healthy control group from the PDS in Stockholm. The majority of patients included in this study, came from socio-economically strong areas (59%). A general dentist assessed the need for specialist dental treatment whilst a paediatric dentist determined the need for GA. Inclusion criteria for the treatment group were need for treatment under GA because of severe caries, in combination with dental fear or behaviour management problems. Exclusion criteria were chronic medical disorders or need for GA due to oral surgery. During 2006–2007, the Department of Paediatric Dentistry at The Eastman Institute treated 297 patients under GA. Of these, 226 were excluded due to chronic medical disorders or oral surgery. The final treatment group comprised 71 healthy patients (44 boys and 27 girls) with a mean age of 8.1 years (3–18 years). The healthy control group (with no chronic medical disorders, n = 29) came from the same public clinics as the referred patients. To build a control group, each patient in the treatment group were matched with three patients at the same PDS clinic who had not been referred to the Department of Paediatric Dentistry, Eastman Institute. The control group comprised 213 patients (132 boys and 81 girls) with a mean age of 8.1 years (3–18 years, Fig. 1).

Two of the authors (GT and JP) extracted all data from the electronic patient records (T4 Practice Management Software, CareStream Dental AB). For the treatment group, data on the number of decayed primary and permanent teeth, as well as treatment under GA, came from patient records at the Department of Paediatric Dentistry, Eastman Institute whilst data on dental treatments and missed appointments before the referral came from patient records from the PDS between the baseline period (1999–2001) and the date of referral (between 2003 and 2007). The reason for the 3-year baseline period is that the introduction of the electronic patient records took 3 years (1999–2001). For both the treatment and control groups, recordings were made of the number of missed appointments; number of cancelled appointments; introductions to treatment; number of prophylactic treatments; number of visits to the dentist, dental nurse, or dental hygienist; number of dentists during the treatment period; number of conscious sedations with midazolam; restorations; extractions; fissure sealants; bite-wing radiographs; and decayed teeth in the primary and permanent dentitions (dt and DT).

### Statistical analysis

All statistical calculations used a software package (IBM SPSS Statistics 21.0). The Mann–Whitney U test compared the treatment and control groups according to the distributions of numerical variables. A Chi square test compared the socio-economic backgrounds of the GA group and control group. Stepwise logistic regression analysis calculated the impact of all potential explanatory factors on the dependent variable “treatment under general anaesthesia.” Results were statistically significant at *p* < 0.05.

## Results

No significant difference was found in socio-economic background between the treatment and control groups. Tables 1, 2, 3 and 4 show the patient history data before referral to the Department of Paediatric Dentistry.

**Table 1:** Means (range) of investigated variables for treatment and control groups before referral to paediatric dentistry

Variable	Treatment group (N = 71)	Control group (N = 213)	*p*
Age (years)	8.1 (3–18)	8.1 (3–18)	NS
Number of primary teeth with caries	5.2 (0–16)	0.49 (0–12)	<0.001
Number of permanent teeth with caries	0.73 (0–7)	0.07 (0–3)	<0.001
Number of dental visits	4.9 (0–16)	3.0 (0–17)	<0.001
Number of dentists during the period prior to referral	2.0 (0–5)	1.4 (0–6)	<0.001
Number of dental nurse visits	0.66 (0–10)	0.51(0–6)	NS
Number of dental hygienist visits	0.80 (0–4)	0.54 (0–4)	NS
Number of missed appointments	0.54(0–5)	0.42 (0–6)	NS
Number of cancelled appointments	0.17 (0–3)	0.43 (0–7)	<0.05
Number of visits for behaviour management treatment	0.65 (0–7)	0.02 (0–1)	<0.001
Number of visits for preventive treatment	1.0 (0–6)	0.23 (0–4)	<0.001
Number of conscious sedations with midazolam	0.23 (0–2)	0.02 (0–1)	<0.01
Number of restorations	1.8 (0–13)	0.83 (0–12)	<0.01
Number of tooth extractions	0.18 (0–4)	0.17 (0–3)	NS
Number of teeth with fissure sealant	0.01 (0–1)	0.08 (0–4)	NS
Number of bite-wing radiographs	1.9 (0–10)	2.0 (0–13)	NS

**Table 2:** Description of the number of visits and nature of treatment in preschool children (3–6 years), school-age children (7–12 years), and adolescents (13–18 years) in the treatment and control groups prior to referral to the specialist clinic

Treatment variables (n = number of)	3–6 years (N = 120)		7–12 years (N = 136)		13–18 years (N = 28)	
	Treatment group (N = 30)	Control group (N = 90)	Treatment group (N = 34)	Control group (N = 102)	Treatment group (N = 7)	Control group (N = 21)
Decayed primary teeth	6.1 (0–16)***	0.2 (0–5)***	5.3 (0–15)***	0.8 (0–12)***	0.4 (0–3)	0.4 (0–3)
Decayed permanent teeth	0	0.01 (0–1)	1.1 (0–4)***	0.1 (0–3)***	2.1 (0–7)*	0.2 (0–2)*
Dental visits	3.3 (0–9)***	0.9 (0–7)***	6.7 (1–16)**	4.1 (0–17)**	3.4 (0–6)*	6.7 (2–12)*
Dentists during the period prior to referral	1.8 (0–4)***	0.7 (0–5)***	2.3 (1–5)	1.9 (0–6)	1.6 (0–3)	2.2 (1–6)
Dental nurse visits	0.5 (0–3)	0.4 (0–4)	0.8 (0–10)	0.7 (0–6)	0.4 (0–2)	0.4 (0–3)
Dental hygienist visits	1.1 (0–4)	0.7 (0–3)	0.7 (0–3)	0.5 (0–4)	0	0.1 (0–1)
Missed appointments	0.3 (0–5)	0.2 (0–3)	0.7 (0–5)	0.6 (0–6)	0.9 (0–3)	0.6 (0–3)
Cancelled appointments	0.2 (0–3)	0.3 (0–6)	0.1 (0–1)*	0.5 (0–7)*	0.3 (0–2)	0.6 (0–3)
Visits for behaviour management treatment	0.57 (0–4)***	0***	0.8 (0–7)***	0.04 (0–1)***	0.1 (0–1)	0
Visits for preventive treatment	1.0 (0–5)***	0.1 (0–2)***	1.1 (0–6)***	0.3 (0–2)***	0.3 (0–1)	0.4 (0–4)
Restorations	0.8 (0–5)***	0.1 (0–5)***	2.8 (0–13)	1.2 (0–12)*	1.4 (0–3)*	2.0 (0–7)
Extraction	0.03 (0–1)	0.02 (0–1)	0.3 (0–4)	0.3 (0–3)	0	0.3 (0–2)
Teeth with fissure sealant	0	0	0.03 (0–1)	0.1 (0–4)	0	0.2 (0–3)
Radiographic examination (BW)	1.2 (0–10)**	0.2 (0–2)**	2.3 (0–9)	2.5 (0–9)	2.1 (0–6)**	7.0 (0–13)**

*BW* Bitewing radiograph

*p < 0.05, **p < 0.01, ***p < 0.001

**Table 3:** Frequency distribution of visits for behaviour management treatment in the treatment and control groups

Number of visits	Treatment group (N)	Control group (N)
0	46	209
1	16	4
2	4	0
3	1	0
4	3	0
5	0	0
6	0	0
7	1	0

**Table 4:** Frequency distribution of visits for preventive treatment in the treatment and control groups

Number of visits	Treatment group (N)	Control group (N)
0	34	179
1	19	22
2	10	11
3	5	0
4	0	1
5	2	0
6	1	0

### Number of dental visits

Overall, patients in the treatment group visited the dentist significantly more often (*p* < 0.001) and saw significantly more dentists during the treatment period (*p* < 0.001) than the control group. There were no significant differences between the groups in number of visits with a dental nurse or dental hygienist. Neither did the number of missed appointments differ significantly. The number of cancelled appointments, however, were significantly fewer (*p* < 0.05) in the treatment group (Table 1).

Preschool (aged 3–6 years, *p* < 0.001) and school-age (aged 7–12 years, *p* < 0.01) children in the treatment group visited the dentist significantly more often than those in the control group. This differed from the adolescents (aged 13–18 years) in the treatment group, who had significantly fewer visits to the dentist (*p* < 0.05) compared to the control group. There were no significant differences in visits to the dental nurse or dental hygienist between the various age groups in the treatment and control groups.

No significant differences were found between the groups in missed or cancelled appointments in any age group, except amongst school-age children in the control group, who cancelled their appointments significantly more often than school-age children in the treatment group (Table 2).

### Number of visits for behaviour management

Overall, patients in the treatment group had 0.65 visits for behaviour management compared to 0.02 visits for the control group (*p* < 0.001, Table 1). Preschool and school-age children in the treatment group had significantly more visits for behaviour management (*p* < 0.001) than the control group (Table 2). When looking at the distribution of visits for behaviour management, 65% of the treatment group had no visits and 23% had only one visit (Table 3).

### Number of visits for preventive treatment

Overall, patients in the treatment group made 1.0 visits for preventive treatment compared to 0.23 visits in the control group (*p* < 0.001, Table 1). Preschool and school-age children in the treatment group visited their clinic significantly more often for preventive treatment (*p* < 0.001) than those in the control group (Table 2). In terms of visits for preventive treatment, 48% of the treatment group had no visits, 27% had only one visit, and 14% had two visits (Table 4).

### Number of decayed teeth

Overall, the mean number of decayed teeth was significantly higher in the treatment group (*p* < 0.001) than in the control group, in both the primary as well as the permanent dentition (Table 1). Preschool children had a mean number of 6.1 decayed primary teeth and 0 decayed permanent teeth, school-age children had 5.4 decayed primary teeth and 1.1 decayed permanent teeth and adolescents had 0.4 decayed primary teeth and 2.1 decayed permanent teeth. In preschool and school-age children in the treatment group, the mean number of decayed teeth was significantly higher (*p* < 0.001) than for those in the control group (Table 2).

### Treatment variables

Overall, the treatment group received significantly more treatments under sedation with midazolam (*p* < 0.01) and had significantly more restorations (*p* < 0.01) than the control group (Table 1). No significant differences between the two groups in extractions, fissure sealants, or number of bite-wing radiographs.

Preschool (*p* < 0.001) and school-age (*p* < 0.05) children in the treatment group had significantly more restorations than those in the control group, contrary to the adolescents who had significantly fewer restorations (*p* < 0.05) than their counterparts in the control group (Table 2).

Table 5 presents the results of the final part of the stepwise logistic regression analysis. Visits for behaviour management (*p* < 0.01) and preventive (*p* < 0.05) treatments occurred significantly more often in the treatment group. In addition, treatment under GA was significantly more common in children with many carious teeth (*p* < 0.001) and those with fewer restorations (*p* < 0.05).

**Table 5:** Results of stepwise logistic regression analysis with the decision to perform treatment under general anaesthesia as the dependent variable

Variable (n)	Odds ratio	Confidence interval	*p*
Introductions to treatment	10	(2.3; 45)	< 0.01
Prophylactic treatments	1.8	(1.1; 3.0)	< 0.05
Teeth with caries	2.2	(1.7; 2.7)	< 0.001
Restorations	0.75	(0.56; 0.98)	< 0.05

## Discussion

This retrospective cohort study suggests that children and adolescents with severe dental decay and who were treated under GA may not have received enough preventive treatment or behaviour management before referral to the specialist clinic for paediatric dentistry.

A recently published study demonstrated that caries prevalence (deft and defs) and occurrence of apical periodontitis and infection due to pulpal necrosis were significantly higher amongst preschool children treated under GA than amongst controls. They also made significantly more emergency visits and had had previous treatments under sedation (Kvist et al. 2014). Our study supports earlier findings that children with dental anxiety and/or behaviour management problems treated under GA have higher rates of caries (Smallridge et al. 1990; Adams and Landes 2005; Macpherson et al. 2005; Torriani et al. 2014; Dahlander et al. 2015). Both children and adolescents in our treatment group showed significantly higher frequencies of carious lesions in the primary and permanent dentitions than their counterparts in the control group. Furthermore, the treatment group had significantly more treatments under sedation with midazolam and significantly more restorations.

The present study found no significant difference in missed appointments between the two groups and significantly fewer cancelled appointments in the treatment group. These findings are in contrast with previous studies that show a higher frequency of dental avoidance amongst children and adolescents with dental anxiety and behaviour management problems (Klingberg et al. 1994; Skaret et al. 1998; Wigen et al. 2009). It may be that children and adolescents in the treatment group, with the support of their parents, are more motivated to have dental treatment because they have substantial dental decay.

Behaviour management is a key factor when treating children and adolescents with dental fear and/or dental anxiety. If the behaviour of the child or adolescent in the dental surgery/office cannot be managed, it is very difficult, and sometimes impossible, to provide the necessary dental care. In addition, parental influences play a major role in how the child or adolescent manages the stress of dental treatment. The present study found that preschool and school-age children in the treatment group received significantly more behaviour management than their counterparts in the control group, but not adolescents, who showed no significant difference. However, 65% (n = 46) of the treatment group received no behaviour management and 23% (n = 16) had only one visit for behaviour management before referral to the paediatric dentistry clinic.

Diercke et al. (2012) showed that general dentists use behaviour management techniques less frequently than paediatric dentists and that the majority of dentists reported difficulty treating children with dental anxiety. One factor that may play an important role in this is the lack of continuing education amongst general dentists about treating children and adolescents with dental anxiety and/or behaviour management problems. Studies have shown that a high proportion of general dentists do not attend post-graduate courses in behaviour management techniques (Diercke et al. 2012; Strøm et al. 2015). It is important that the general dentist, and the dental team as a whole, have continuing education in behaviour management of children and adolescents. The aim is to achieve a relationship with both the parents and the child that makes it possible to deliver dentistry to the highest possible standards. Furthermore, it is important to help every child develop the skills and behaviours to cope with dental care without anxiety or fear.

Despite improvements in children’s oral health, children living in areas of low socio-economic status still experience a significant disease burden from dental caries (Källestål and Fjelddahl 2007; Stecksén-Blicks et al. 2008; Alm et al. 2012; Dahlander et al. 2015). Early intervention not only prevents progression of caries in the primary dentition but also reduces caries prevalence in the permanent dentition (Skeie and Klock 2014). Special efforts to prevent disease development in children living in areas characterised by low socio-economic status have been unsuccessful (Anderson et al. 2016). Interventions must start at an early age, before the onset of dental caries, and it is important to educate parents to become competent in managing daily preventive measures (Aljafari et al. 2014). An international study comparing inequalities in childhood caries found that parents’ perception of their own ability to control their child’s tooth brushing and sugar intake was the most important factor in establishing favourable oral-health behaviour (Pine et al. 2004). Parental beliefs and attitudes seem to play an important role in moderating oral health-related behaviour in young children and in determining whether they will develop caries.

In healthy children and adolescents treated under GA due to severe tooth decay, preventive treatment has been unsuccessful (Karki et al. 2011). That study demonstrates that preschool and school-age children in the treatment group had received significantly more preventive care than the control group, which was in contrast to adolescents, where there was no significant difference between the groups. Interestingly, when looking at the distribution of preventive treatment, 48% (n = 34) had no visits for prevention and 27% (n = 19) had only one visit for preventive treatment before referral to the Department of Paediatric Dentistry. These results may indicate a failure in risk assessment and preventive treatment in this high-risk caries group. Previous studies demonstrating that preventive treatment does not always correlate with a patient’s need tends to support our finding (Helminen and Vehkalahti 2003).

The stepwise logistic regression analysis showed that the most significant factor affecting the choice of sedation with GA was number of decayed teeth. Furthermore, there was a significantly greater tendency for dental treatment under GA if the patient had received more behaviour management and preventive treatment.

One has to consider that there are limitations of this study. The small numbers of subjects included as well as the retrospective design makes it difficult for determining any temporal relationships.

## Conclusion

In the Stockholm PDS, over half of the children and adolescents referred by general dentists to paediatric specialists had no behaviour management treatment and nearly half, had received no preventive treatment, despite receiving significantly more operative treatment compared with matched controls. General dentists should target high caries-risk patients for additional behaviour management and preventive care to reduce the need for treatment under GA.
